# isomiR-SEA: an RNA-Seq analysis tool for miRNAs/isomiRs expression level profiling and miRNA-mRNA interaction sites evaluation

**DOI:** 10.1186/s12859-016-0958-0

**Published:** 2016-03-31

**Authors:** Gianvito Urgese, Giulia Paciello, Andrea Acquaviva, Elisa Ficarra

**Affiliations:** Department of Control and Computer Engineering DAUIN, Politecnico di Torino,, C.so Duca degli Abruzzi 24, Turin, 10129 IT Italy

**Keywords:** miRNA, isomiR, miRNA-mRNA interaction site, Expression level

## Abstract

**Background:**

Massive parallel sequencing of transcriptomes, revealed the presence of many miRNAs and miRNAs variants named isomiRs with a potential role in several cellular processes through their interaction with a target mRNA. Many methods and tools have been recently devised to detect and quantify miRNAs from sequencing data. However, all of them are implemented on top of general purpose alignment methods, thus providing poorly accurate results and no information concerning isomiRs and conserved miRNA-mRNA interaction sites.

**Results:**

To overcome these limitations we present a novel algorithm named isomiR-SEA, that is able to provide users with very accurate miRNAs expression levels and both isomiRs and miRNA-mRNA interaction sites precise classifications. Tags are mapped on the known miRNAs sequences thanks to a specialized alignment algorithm developed on top of biological evidence concerning miRNAs structure. Specifically, isomiR-SEA checks for miRNA seed presence in the input tags and evaluates, during all the alignment phases, the positions of the encountered mismatches, thus allowing to distinguish among the different isomiRs and conserved miRNA-mRNA interaction sites.

**Conclusions:**

isomiR-SEA performances have been assessed on two public RNA-Seq datasets proving that the implemented algorithm is able to account for more reliable and accurate miRNAs expression levels with respect to those provided by two compared state of the art tools. Moreover, differently from the few methods currently available to perform isomiRs detection, the proposed algorithm implements the evaluation of isomiRs and conserved miRNA-mRNA interaction sites already in the first alignment phases, thus avoiding any additional filtering stages potentially responsible for the loss of useful information.

**Electronic supplementary material:**

The online version of this article (doi:10.1186/s12859-016-0958-0) contains supplementary material, which is available to authorized users.

## Background

miRNAs are nowadays recognized as a class of evolutionally conserved, single-stranded, 19–23 nucleotides (nt) long non-coding RNAs acting, both in plants and animals, as post-transcriptional regulators. Genes codifying for miRNAs can be localized at different sites into the genome even if, in humans, the majority of them have been detected into the transcripts intronic regions. The involvement of miRNAs in almost every physiological process such as cell fate determination, proliferation, cell death and in many cellular activities among which immune response, insulin secretion, neurotransmitter synthesis, circadian rhythm and viral replication is widely proven [1–4]. Furthermore, recent researches have shown deregulated miRNAs expression in many pathologies such as cardiovascular diseases [5], autoimmune disorders [6] and cancers with implications in numerous oncogenic or tumor suppressor pathways [7–9].

Recently, thanks to the advent of Next Generation Sequencing (NGS) technologies, it is possible to carefully detect and quantify the amount of both known and novel miRNAs in RNA-Seq samples, accounting also for single nucleotide polymorphisms (SNPs) [10–13]. The high quality of data coming from NGS technologies has been exploited to gain novel insights into miRNAs variants called isomiRs. isomiRs are miRNAs variants that originate from miRNAs loci as consequence of specific processes as exoribonucleases or nucleotidyl transferase activity, RNA editing, SNPs or imprecise cleavage by the ribonucleases Drosha and Dicer [14]. Accordingly to Neilsen and colleagues research [15], isomiRs can be distinguished into three main classes: 3’ isomiRs, 5’ isomiRs and polymorphic (SNP) isomiRs. Specifically the first have been identified in different studies [16–18] as the most common and abundant variants in both plants and animals. Recently the functionality of 3’ isomiRs has been investigated with regard to compensatory base pairing at the 3’ end [19] and distinct silencing functions attributed to different miRNA family members with shared seed sequences but divergent 3 ends [20]. Fernandez and colleagues [21] proven the variation of isomiRs expression levels in Drosophila Melanogaster, depending on the developmental stage and the specific tissue, thus suggesting a role for these miRNAs variants in different biological processes. In 2012 Li and colleagues [22] pointed out different isomiRs expression patterns in normal and tumour gastric tissues calling for isomiRs relevance during miRNAs analyses. Moreover, results from these studies seem to account for an impact of isomiRs on target selection, miRNA stability or a different loading into the RISC complex. In particular, with respect to isomiRs target selection, evidences of an impact of miRNA sequence variations on target mRNA selection have been recently experimentally validated by Tan and colleagues [23].

**Fig. 1:**
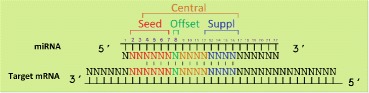
Interaction sites Scheme. Reported, respectively with red, green, blue and brown colors, the possible miRNA-mRNA interaction sites known as *seed (nt 2–7)*, *offset (nt 8)*, *supplementary (13–16)* and *central (nt 3–16)*

Both miRNAs and isomiRs act at the post transcriptional level via base-pairing with complementary sequences on target mRNAs. As a result, the target mRNA molecules are silenced by one of the following mechanisms: i) cleavage of the mRNA strand, ii) destabilization of the mRNA through the removal of its poly(A) tail, or iii) translation of the mRNA into proteins by ribosomes. The capability of a miRNA in bounding a specific target depends on the conservation, onto the miRNA structure, of particular nucleotide sub-sequences called miRNA-mRNA interactions sites, among which of fundamental importance the seed sequence (red sequence in Fig. 1). However, additional miRNA-mRNA interactions sites have been recently identified in Bartel researches [2, 24] as able to guarantee high specificity in miRNA-mRNA interaction and have been adopted as central features by the most of the target prediction tools to avoid false discoveries [25–27].

Many software tools have been developed during the last decade to extract miRNAs expression levels from RNA-Seq data. Among them, miRDeep [28, 29], miRanalyzer [30, 31], miRExpress [32], miRTRAP [33], DSAP [34], mirTools [35], MIReNA [36], miRNAkey [37] and mireap [38] have been recently compared showing quite different performances in terms of sensitivity and accuracy levels as well as poorly overlapping miRNAs output sets [39]. Being developed on top of general purpose alignment algorithms as Bowtie [40] or BWA [41], these tools are not able to evaluate the encountered mismatches accordingly to their positions on miRNA sequence. This accounts for reduced sensitivity and accuracy levels in miRNAs detection and for their incapability in distinguishing between the different isomiRs and modifications in miRNA-mRNA interaction sites. For instance, a complete and uncorrupted seed sequence is fundamental to ensure the miRNA:mRNA complex stability [2]. Differently from state of the art tools, isomiR Seed Extension Aligner (isomiR-SEA) exploits a miRNA specific alignment algorithm, able to correlate the encountered mismatches with their positions on the miRNA sequence and then to produce accurate miRNAs expression level quantification and both isomiRs and miRNA-mRNA interaction sites precise classification. All this information is collected during the alignment procedure, thus avoiding additional filtering stages potentially responsible for the loss of useful data. It is worth noting that the evaluation of the impact of isomiRs on the conserved miRNA-mRNA interaction sites has two main consequences. On the one hand it allows to clarify the impact of isomiRs on miRNAs functionalities thus gaining novel insights into miRNAs regulative behaviour. On the other hand, on the basis of the observed results, it is possible to decide whether to include or not detected isomiRs in the calculated miRNAs expression level.

In the “*Implementation*” Section additional algorithmic implementation details are provided. isomiR-SEA performances, assessed on two different public datasets, will be presented and discussed in the “*Results and discussion*”. This section is organized as follows: The details concerning the experimental set-up adopted to perform the analysis are given in the “*Testing procedure*” Subsection. In “*isomiR-SEA isomiRs and interaction sites detection*” Subsection are provided results obtained on the first dataset, that highlight the variety of information extracted by isomiR-SEA. The comparisons, in terms of computed expression levels between isomiR-SEA and two state of the art tools, performed on the second dataset, are shown in *isomiR-SEA comparisons*. Finally in “*Computational costs*” the computational costs proper of the three tools are discussed. In the “*Conclusions*” Section the final remarks deriving from the proposed work are highlighted.

## Implementation

The isomiR-SEA tool exploits a miRNA-tailored alignment procedure, named miR-SEA [42], that implements an accurate miRNA model derived from experimental evidences [2]. The model is built upon the main features characterizing the *seed* sequence (red sequence in Fig. 1), which is nowadays recognized to play a fundamental role in target recognition and, as consequence, in genes regulation [43, 44]. isomiR-SEA keeps track of the results achieved at each step of the alignment procedure and analyses them in order to recognize miRNAs variants and characterize miRNA-mRNA interaction sites.

**Fig. 2:**
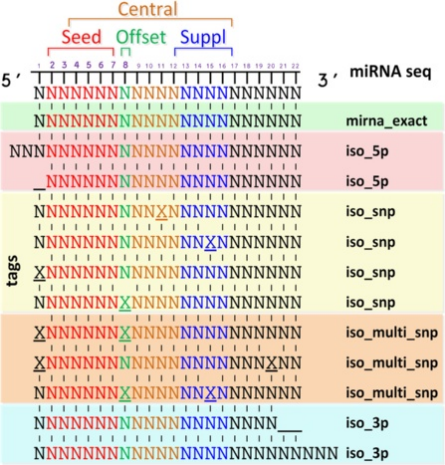
isomiRs Scheme. In Figure are reported the different miRNAs variants, namely isomiRs, detected by isomiR-SEA. Tags exactly matching a miRNA (mirna_exact) identify a miRNA molecule, those with 5p (iso_5p), 3p (iso_3p), snp (iso_snp) or multi snp (iso_multi_snp) variations, respectively isomiRs 5p, 3p, snp and multi-snp

The tool is designed to distinguish among four miRNA variations with respect to the exact miRNA sequence (*mirna_exact*). As shown in Fig. 2 isomiR-SEA is able to classify the aligned tags as: i) *mirna_exact*; ii) *iso_5p* resulting from insertion or deletion in the 5p-end; ii) *iso_snp* or *iso_multi_snp* harbouring one or more mismatches wherever in their sequences (included the 3p and 5p-ends); iv) *iso_3p* arising from insertions or deletions in the 3p-end.

As a consequence of the addition or deletion of nucleotides and the presence of SNPs, remarkable effects on miRNAs target selection can be observed [23]. Then the conservation in the tag sequence of miRNA-mRNA interaction sites is checked during isomiR-SEA alignment procedure. Figure 1 reports on the four interaction sites evaluated by isomiR-SEA: i) The *seed* sequence, a region located at nucleotides 2–7 shared by all the miRNAs belonging to the same family and known to be the most important interaction site between miRNA and its mRNA targets; ii) the *3’-supplementary site*, a sequence corresponding to nucleotides 13–16 on the miRNA structure and characterized as sensitive to pairing geometry preferring at least 3–4 contiguous Watson-Crick pairs not interrupted by bulges, mismatches or wobbles; iii) the *central site*, a region on the miRNA corresponding to nucleotides 4–16 and iv) the *offset site*, at nucleotide 8 on the miRNA sequence. Moreover, in order to further improve the accuracy of the calculated expression levels and to correctly distinguish among miRNAs belonging to the same family, ambiguously mapped tags (i.e. tags assigned to more than one miRNA), are evaluated on the basis of their alignment scores and assigned to a unique miRNA.

**Fig. 3:**
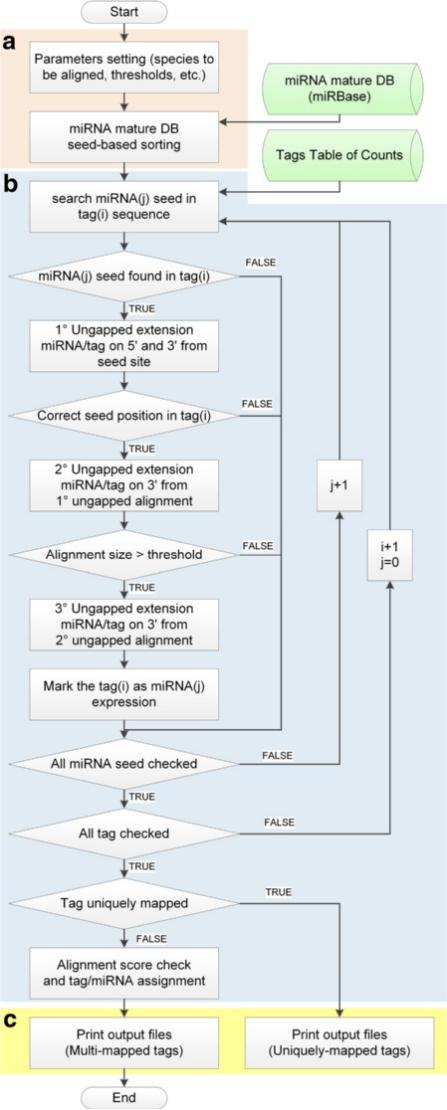
isomiR-SEA work-flow. In Figure are reported the three main blocks which constitute isomiR-SEA. Block (**a**) reports on parameters setting and input files preprocessing, block (**b**) on the alignment procedure and finally block (**c**) on the output files generation

From an implementation view point, the accurate miRNA model proper of isomiR-SEA guarantees the deletion of those tags not compatible with miRNAs structure, thus reducing the computational costs required for the following steps of the pipeline. In particular, those tags that show an incomplete or corrupted or shifted seed sequence are removed. Finally, isomiR-SEA standalone C ++ implementation supported by SeqAn library [45], makes the proposed pipeline user-friendly and easy to customize. Specifically, isomiR-SEA algorithm starts with the search into the tags for miRNAs seeds located in positions compatible with miRNA structure to discard sequences not accounting for miRNAs molecules. The remaining tags are progressively extended in 3’ direction allowing for mismatches. Mismatches positions are evaluated during each performed extension to check whether or not occurring within a selected nucleotide interval. Tags satisfying this last criterion are divided into two groups on the basis of their attribution to a single or multiple miRNAs. Sequences belonging to the second group are scored using specific alignment parameters and finally assigned to a specific miRNA (details in the “Implementation” Section, Subparagraph B). Finally isomiRs and interaction sites are annotated by evaluating the mismatches detected during tags alignments. The whole work-flow is basically constituted of the three main blocks depicted in Fig. 3 with letters a, b and c. Each of them will be detailed in the following.

### A. Parameters setting and input files pre-processing

Twelve parameters allow users to trigger isomiR-SEA behaviour. Specifically, tags can be aligned on one or more species by imposing a minimum alignment length and the start/end positions where the seed has to be searched into the tag sequence. Moreover users can decide which alignments to discard by changing the value of a specific parameter introduced to evaluate miRNA-tag identity.

Two input files have to be provided to isomiR-SEA tool for the detection of miRNAs expression levels as shown in Fig. 3a. The first file contains miRNAs reference sequences while the second stores the input tags table of counts (i.e. table reporting the occurrence in the sample of each tag) on which the analysis has to be carried out (green cylinders in Fig. 3).

miRNAs reference sequences have been downloaded from miRBase [46], a well known miRNAs repository currently at version 21. The file containing miRNAs sequences, downloaded from miRBase database, has to be processed in the so called *mature DB seed-based sorting* step, where all the seeds are firstly extracted from the miRNAs reference sequences, then classified depending on the species and finally grouped together. The obtained groups, sharing the same seed sequence, are then stored in an optimized data structure allowing its efficient access during the alignment procedure.

The second input file is the standard tags counts file. Generally this file is obtained by means of freely available tools performing the following steps: i) Trimming of adapter sequences on raw *fastq* or *fasta* reads, output of sequencing machines; ii) grouping in unique sequences called *tags*; iii) calculation of the occurrence of reads supporting the various tags. The output file, reporting tag sequences followed by their occurrence, is stored by isomiR-SEA in the main memory.

### B. isomiR-SEA alignment procedure

Each miRNA seed is searched, in this phase, into each stored tags (as reported in block b of Fig. 3). Tags harbouring the seed are extended without gaps allowed in both directions (ungapped extension). This procedure is done for all the miRNAs sharing the same detected seed in a position compatible with miRNA structure [2]. Tags not satisfying these conditions are currently discarded and will be not considered in the further steps of the analysis increasing, in this way, the computational speed. As future work, we are planning to re-map these tags on pre-miRNA sequences by adopting an accurate local aligner implementing *DGS* model [47].

The selected tags are further extended in 3’ direction allowing for the presence of a second mismatch. At this point, the distance of the second encountered mismatch with respect to the previous found, is evaluated. Tags characterized by two mismatches in the first 10 aligned nucleotides are discarded, whereas the others are further extended until the end of the alignment or a third mismatch. This threshold parameter can be modified by the user. However the default value has been chosen according to experimental insights [2].

Finally, each aligned tag is scored as reported in Eq. 1 where *miR*_*size*_ is the length of the miRNA on which the tag has been aligned, *#gaps* the number of gaps detected in the alignment and *align*_*size*_ the length of miRNA-tag alignment. Thanks to this measure it is possible to evaluate the quality of the different alignments. Lower values are associated to better alignments.

(1) $$ align_{score}= \frac{miR_{size}-(align_{size}-\#gaps)}{miR_{size}}100   $$

Furthermore, to take into account also the tag length (*tag*_*size*_) during tags alignment assessment, a second measure has been introduced as shown in Eq. 2. The lower *miRtag*_*diff*_, the higher the comparability between the tag and the miRNA.

(2) $$ miRtag_{diff}= miR_{size}-tag_{size}   $$

These two scores can be adopted to select the most reliable alignment in case of tags ambiguously assigned to more than a miRNA.

As last step, the isomiRs, as well as the interaction sites, are annotated according to the position of the mismatches found during the alignment procedure.

### C. isomiR-SEA output files generation

Nine output files are generated at each isomiR-SEA run in order to allow users to deeply investigate the performed alignments. Output files provide information both from a tag and from a miRNA view point.

In particular, if a tag has been mapped on a unique miRNA its information will be stored in a unique tag file, while if the tag aligned on more than a miRNA its data will be reported in a specific file storing the so called ambiguous tags. Some of these ambiguous tags will be then re-evaluated using the quality parameters (*align*_*score*_ and *miRtag*_*diff*_) and assigned to a unique miRNA. These tags are then stored in a third file.

These tag-related files provide users with all the isomiRs and miRNA-mRNA interaction sites detected starting from the same tag, the unique or multiple miRNAs on which the tag has been mapped and the two mapping parameters values previously described in Eqs. 1 and 2. Furthermore, in case of multi-mapped tags, indications relative to the different detected miRNAs are reported, such as the number of families to which they belong, the number of miRNAs on which the tag has been mapped and the scoring distance of the second best alignment detected from the best one reported.

From a miRNA point of view, two main outputs have been adopted to summarize the detected expression levels, according to the format used by the most known miRNAs discovery tools. The first file aggregates for the different miRNAs the information collected from the unique mapped tags whereas the second file, derived from the selected tags, reports on those tags that have been re-evaluated thanks to the alignment score parameters. Even in this case the information related to isomiRs and interaction sites is reported, together with data concerning the number of supporting reads and the computed alignment scores.

It is worth noting however that users are free to either consider only the miRNAs expression files to acquire the overall expression of the detected miRNAs and isomiRs, or to look at the tags files introducing the preferred filtering stages or grouping data according to their needs.

Concerning the implementation, isomiR-SEA tool has been developed in C++ programming language, taking advantage of the open source SeqAn library [45]. This library provides users with algorithms and data structures for the analysis of biological sequences that ensure high performances and, at the same time, ease of integration in analysis pipelines.

## Results and discussion

We report here the results of isomiR-SEA performance assessment on two datasets extensively analysed in previous researches by Somel and colleagues [48] [GEO:GSE18069] and Li et al. [49] [GEO:GSE31617, GEO:GSE24952] for which miRNAs and mRNAs sequencing data have been freely provided on GEO database repository [50]. These two studies proven respectively the regulative roles of miRNAs in gene expression modulation throughout the entire life span of both humans and macaques and the general or cancer-specific involvement of different genes and miRNAs in the carcinomas of bladder, kidney and testis.

The experiments performed allowed us to show the capability of isomiR-SEA tool in providing a detailed picture of miRNAs, isomiRs and conserved miRNA-mRNA interaction sites characterizing the samples under investigation.

Finally isomiR-SEA performances have been compared with two state of the art tools, i.e. miRanalyzer [30, 31] and miRExpress [32]. In the comparison, both miRNAs expression levels quality and computational times have been evaluated.

### Testing procedure

Both datasets have been analysed by: i) Adopting miRNAs reference databases, the miRBase release 11 (characterized by 6211 mature miRNAs from 74 species), used in the aforementioned works, and the latest release 21 that stores 35828 mature miRNAs sequences from 223 species; ii) using as an input the datasets reads trimmed allowing 2 or 3 mismatches in the adapter sequence; iii) setting the isomiR-SEA running parameters, i.e. seed length and minimum alignment threshold, respectively to 6 (from nt 2 to 7) and 11. In the following, where not explicitly stated, the reported results were obtained using human miRBase 21 as reference. Furthermore results reported in this section have been produced from reads trimmed allowing a maximum number of mismatches equal to 3 and collapsed to get the tags table of counts. Further details are reported in Additional file 1: Text S1.

### isomiR-SEA isomiRs and interaction sites detection

Concerning Somel dataset, miRNAs reads were collected from the superior frontal gyrus of 12 healthy humans and 12 rhesus macaques of different ages, selected in a cohort of individuals. In agreement with previous Somel et. al researches [48], we will refer in the following to human *developmental* and *ageing* stage samples, respectively for data collected from <20 years old and >20 years old humans. This threshold has been fixed at 4 years for macaques.

Since the considered dataset includes two different species, in order to perform a more specific evaluation of human miRNAs expression levels, the reads alignment has been performed by adopting as reference the union of human and macaque miRBase sequences. This allowed to discard from the computed human miRNAs expression levels those tags better aligned on macaques miRNAs.

Results shown in the following have been obtained by adopting this experimental set-up and are discussed in this section to highlight trends associated to exact miRNAs, to different isomiRs, conserved miRNA-mRNA interaction sites and overall reads counts. In particular we report the results of the analysis of these behaviours in relation to *ageing* and *developmental* stages in both the species, pointing out the differences and similarities between them where present.

**Fig. 4:**
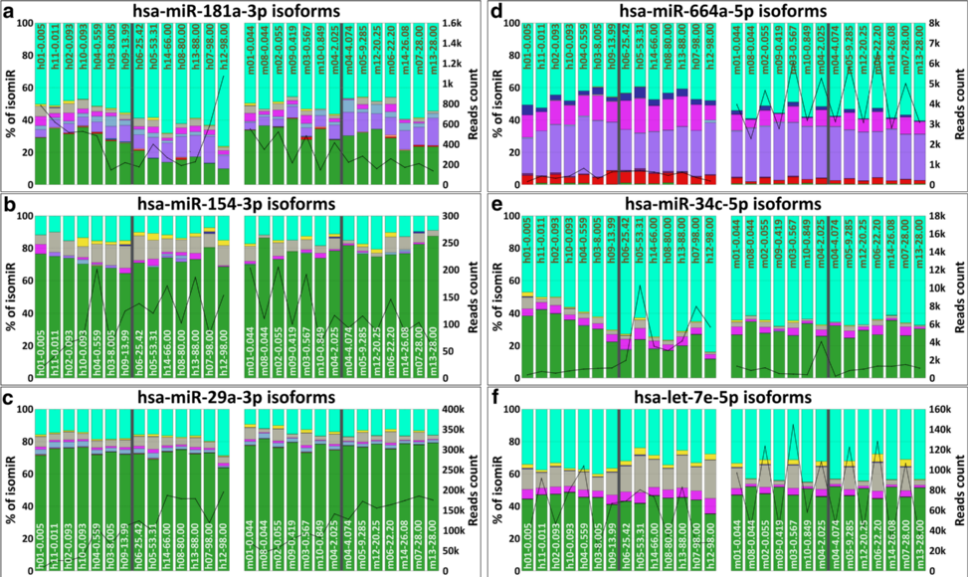
isomiRs percentages. Subfigures (**a**), (**b**), (**c**), (**d**), (**e**) and (**f**) show respectively for human miR-181a-3p, miR-154-3p, miR-29a-3p, miR-664a-5p, miR-34c-5p and let-7e-5p, the isomiRs percentages detected in the samples on x-axis. In particular the left y-axis reports on the percentages of isomiRs, whereas the right y-axis on the absolute reads counts. In each Subfigure the first 14 bars are relative to human samples, whereas the last 14 to the macaque ones. Moreover the *development* and *ageing* stages are divided by a dark-grey bar. The different samples are labelled with a unique identifier in the format *speciesnumsample-age*: The character *h* and *m* identify respectively human or macaque samples, *numsample* ranges from 01–14 for both the species and the age is in the form of years. The bold line, that separates in two groups the analysed samples in both the species, represents the transition age that divides the developmental from the ageing stages. IsomiRs are depicted with different colors according to the legend of Fig. 5. The black trend curve reports on isomiR-SEA reads counts

**Fig. 5:**
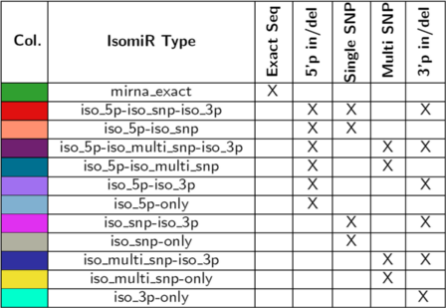
IsomiRs legend. The table reports, for both the tags exactly aligned on miRNA structure or deriving from isomiRs (Column 2), the colors adopted for their representation in the different graphs (Column 1) and the description of the detected mutations (Columns 3–7)

The overall amount of mapped reads and the detailed isomiRs spectrum of six different miRNAs are reported in Fig. 4. These six miRNAs have been selected since characterized by heterogeneous isomiRs and miRNA-mRNA interaction sites spectrum trends that allowed us to widely describe the isomiR-SEA features. Each bar of the six stacked histograms in Fig. 4 describes, for a selected miRNA, the percentage (left y-axis) of read counts assigned by isomiR-SEA to the exact miRNA sequence (%mirna_exact) or to the different detected isomiRs accordingly to the legend reported in Fig. 5. These percentages are computed as the ratio between the read counts attributedby isomiR-SEA to each isomiR type and the total reads count for the specific miRNA. The notation used to identify the different samples on x-axis is in the form *SampleID-age* where *SampleID* contains the character *h* or *m* respectively for human or macaque samples and *age* is expressed in years in order to allow the identification of the transition age from the *developmental* to the *ageing* stage (in Fig. 4 divided by a dark-grey bar). As a consequence, the first seven bars in the stacked histograms represent, for a given miRNA, the associated trends in the *developmental* human brain, whereas the following seven the associated trends in the *ageing* stage. A similar representation stands for the bars related to the macaque samples, for which the transition age has been fixed at four years old. In Fig. 4 groups of samples belonging to the two different species are separated by a wider white bar. The trend curve, depicted in Figure with a black line, reports on the right y-axis read counts for the specific miRNA in the different samples under analysis.

**Fig. 6:**
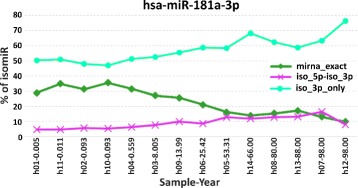
hsa-miR-181a-3p isomiRs trends. In Figure are reported accordingly to the legend of Table 8, the trend lines of *mirna_exact*, *iso_5p-iso_3p* and *iso_3p-only* percentages (y-axis) detected for human miR-181a-3p in the different analysed samples (x-axis)

With reference to **miR-181a-3p** in Fig. 4a it can be observed that the percentage of reads attributed to *iso_3p-only* increases during ageing, reaching a peak of 76.3 % in Sample h12 at 98 years. An analogous trend can be pointed out in relation to *iso_5p-iso_3p* with a maximum of 16.5 % in h07-98.00 while a decreasing trend can be pointed out for *mirna_exact* mapped reads. For the safe of clarity, *mirna_exact*, *iso_3p-only* and *iso_5p-iso_3p* trends are highlighted also in Fig. 6 for miR-181a-3p accordingly to the legend reported in Fig. 5. The analysis of the same miRNA in terms of read counts values is reported in the Additional file 1: Text S2.

**Fig. 7:**
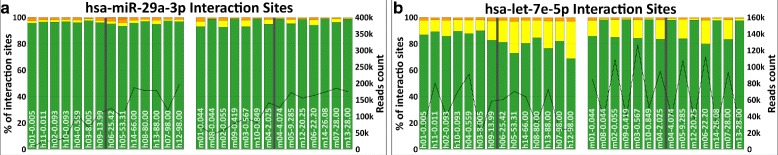
Interaction sites: Subfigures (**a**) and (**b**) report, respectively for hsa-miR-29a-3p and hsa-let-7e-5p, the percentages of tags accounting for the conserved interaction sites on the left y-axis and the absolute reads counts on the right y-axis. Each interaction sites is coloured according to the legend reported in Fig. 8. The first 14 bars are relative to human samples, whereas the last 14 to the macaque ones. Each sample is labelled with a unique identifier in the format species_numsample-age: The character h and m identify respectively human or macaque samples, numsample ranges from 01 to 12 for both the species and the age is in the form of years. The bold line, that separates in two groups the analysed samples in both the species, is located at the transition age. The black trend curve reports on isomiR-SEA reads counts

The account of isomiR types and their percentage is important in order to check the impact of the detected mismatches in the stability of miRNA-mRNA interaction sites (as it will be shown in few examples). These mismatches can compromise the effectiveness of miRNA activity. After this evaluation, the user can decide to discard those tags supporting isomiRs that can lead to unstable or weak interactions, thus obtaining a lower miRNA expression count.

Expression trends depending on the life stage are pointed out also in Fig. 4b relatively to **miR-154-3p**: The percentage of *mirna_exact* reads in humans decreases during the developmental stage until a minimum equal to 64.5 % for Sample h09-13.99 at 14 years old and starts growing again during ageing. Furthermore, an interesting behaviour in *iso_snp* can be identified during the different stages: Their percentages grow until the transition point while they become smaller in the ageing stage.

**Fig. 8:**
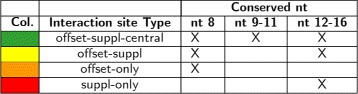
Interaction Sites legend. The table reports, for tags harbouring in their sequences the interaction sites types (Column 2), the colors adopted for their representation in the different graphs (Column 1) and the description of the detected sites (Columns 3–5)

Similar isomiRs percentages in humans and macaques can be detected for **miR-29a-3p**, as highlighted in Fig. 4c. Indeed the overall amount of reads attributed to isomiRs is respectively equal to 27.5 % for humans and 22.5 % for macaques. Somel et al. proved that this miRNA has a role in the strictly developmental regulation in both human and macaque species where it acts by down-regulating cell proliferation-associated genes. Furthermore in humans also cell proliferation proteins appear as down-regulated, strengthening the identified correlation. The analysis of the conserved miR-29a-3p-mRNA interaction sites reported in Fig. 7a can be useful to check if aligned tags, even if related to isomiRs, conserve the miRNA interaction sites and, as a consequence, if the miRNA is able to exploit its function. The representation adopted to describe conserved miRNA-mRNA interaction sites in Fig. 7 is similar to the one reported in Fig. 4: The left y-axis in this case reports the percentages of reads accounting for the different interaction sites detected, the right y-axis reports on the mapped reads counts whereas bar colors describe the 4 different combinations of miRNA-mRNA interaction sites accordingly to Fig. 8. The most of the tags attributed to miR-29a-3p retains all the possible miRNA-mRNA interaction sites accounting for the conservation of miRNA function in down-regulating its target genes coherently to [48].

Coming back to Fig. 4d, it can be seen for **miR-664a-5p**, various isomiRs trends. In particular, human samples are characterized by an higher average percentage equal to 15.1 % of *iso_snp-iso_3p* with respect to macaques, where this percentage is 8.2 %. A similar behaviour, even if less evident, can be also detected for those isoforms classified as *iso_5p-iso_snp-iso_3p*. Furthermore, a relatively small number of *mirna_exact* reads contribute to the total reads number. This isomiRs spectrum is conserved in both species independently from age and developmental stage. The inter-species average values for *mirna_exact* reads are indeed equal to 0.5 % for both humans and macaques.

miR-664a-5p has not been identified by Somel as implicated in regulative processes occurring during development or ageing. The reduced percentages of exact mapped reads provided by isomiR-SEA could explain missed interaction sites and as a consequence justify miR-664a-5p limited regulative role. Two state of the art tools, i.e. miRanalyzer and miRExpress, which performances will be extensively discussed in the following subsection, provided for the considered miRNA an expression level higher than the one calculated by isomiR-SEA: Tags attributed by these tools to miR-664a-5p probably derive from isomiRs and so the produced expression level can not be correctly correlated to miRNA regulative functions.

The percentage of reads attributed to **miR-34c-5p** isomiRs (see Fig. 4e) increases during ageing in humans, whereas a quite stable behaviour can be pointed out in macaques. miR-34c-5p, that has been proven to be differentially expressed during the developmental stage in humans, acts by down-regulating a group of genes involved in neuronal functions [48]. The analysis of the miRNA-mRNA interaction sites in humans, as already observed for miR-29a-3p, highlighted the presence in the tags of almost all the interaction sites explaining miRNA capability in down-regulating target genes consistently with [48]. In fact this miRNA is mainly identified by tags with exact mapping or tags with mismatches in 3p region that do not impact the interaction sites.

Finally, Fig. 4f describes **let-7e-5p** isomiRs spectrum. An increasing percentage of reads attributed to *iso_snp* can be detected in older human samples, whereas quite stable trends are associated to the other identified isomiRs. In order to investigate to what extent miRNAs variations determining isomiRs impact on miRNA-mRNA interactions sites, we report in Fig. 7b the percentages of reads accounting for let-7e-5p conserved interaction sites. A reduction of the percentage of reads attributed to all the three possible interaction modes is observed as age increases. The loss of interaction sites can be attributed to the presence of SNPs in the miRNA region corresponding to the central site thus highlighting a possible role of SNPs in target selection.

### isomiR-SEA comparisons

The second analysed dataset is composed of 27 patient-matched tumor-normal miRNA-Seq samples with ages varying from 30–70. In particular 10 samples derive from Transitional Cell Carcinoma (TCC), 7 from Testicular Germ Cell Tumor (TGCT) and the last 10 from clear cell Renal Cell Carcinoma (ccRCC) [49].

The results obtained by isomiR-SEA on this dataset have been compared to those provided by three state of the art tools, namely miRanalyzer [30, 31], miRExpress [32] and miRDeep2 [28, 29], running with the default configurations. The proposed analysis has been performed in order to show how the strategies implemented by the tools impact on the computation of miRNAs expression levels. In particular, miR-1244 has been selected over the other miRNAs because its alignment results in several isomiRs, and for this reason, miR-1244 is characterized by high variability in the expression levels provided by the various tools. Moreover, its observed low reads counts allowed to easily analyse the tags alignments and highlight the different characteristics of isomiR-SEA and the other two tools.

**Fig. 9:**
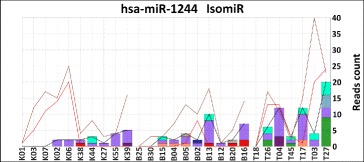
hsa-miR-1244 isomiRs. In Figure are reported for miR-1244 on the y-axis the reads counts associated to the different detected isomiRs in the samples under examination (x-axis). Colors associated to each isomiRs are reported in Fig. 5. Three trend curves account respectively for isomiR-SEA (*black continue*), miRExpress (*red continue*) and miRanalyzer (*brown continue*) reads counts

The miR-1244 tags aligned with the three tools have been extracted and mapped on the miRBase 21. The three compared tools provided quite different results. Figure 9 reports on the y-axis the reads counts provided by isomiR-SEA (black line), miRanalyzer (brown line) and miRExpress (red line) for miR-1244 in the control samples of Li dataset. Analogous considerations can be made for miRNAs expression levels in tumor samples. Furthermore, isomiR-SEA reads counts attributed to miR-1244 and its isomiRs are described in each sample by the bars on x-axis accordingly to the legend reported in Fig. 5 where, as seen in the previous subsection, the bars represent the percentage of tags attributed to a specific isomiR or supporting an exact matching. For the sake of clarity, the expression levels computed by miRDeep2 on a subset of samples from Li dataset are reported in Additional file 1: Text S3.

**Fig. 10:**
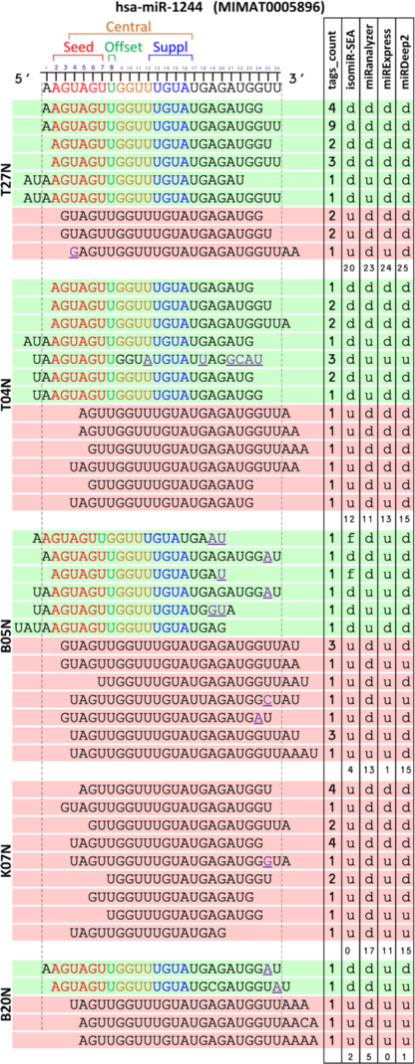
Analysis of isomiR-SEA, miRanalyzer, miRExpress and miRDeep2 mapping results for hsa-miR-1244. In Figure are reported on the left side the tags and their relative occurrence (*right side*) assigned by isomiR-SEA, miRanalyzer, miRExpress and miRDeep2 to miR-1244 in five different samples (T27N, T04N, B05N, K07N and B20N). The *d* character accounts for the detection of the tag, the *u* for its undetection and finally the *f* (only for isomiR-SEA output) for its filtering. The underlined characters identify mismatches in the tag with respect to miRNA sequence. Tags with green background possess a complete seed sequence, whereas those with a red background an incomplete or corrupted seed

Figure 10 shows those tags that have been assigned to **hsa-miR-1244** in five different samples by at least one of the three tools. On the left it is reported the sequence of the aligned tags. Alignments with a green background account for the presence in the tag of the seed sequence, whereas the red ones indicate absence or corruption. The underlined violet characters in each of the aligned tags account for a mismatch with respect to miRNA sequence placed on the top. While the seed, central, offset and supplementary sites have been highlighted only if the tags have been detected by isomiR-SEA. This is because these regions and their isomiRs are annotated by isomiR-SEA only. In the Figure, it is reported also the number of tags supporting the sequence (first column) and the tool that provided the detection. In particular a *d* character indicates the detection of the tag by the considered tool, a *u* character the rejection and finally a *f* letter indicates (only for isomiR-SEA) that the tag has been detected but filtered out during the plotting phase being shorter than a fixed isoform size threshold. The *f* parameter has been adopted to underline that results coming from isomiR-SEA tool can be filtered according to users specific requirements implemented by triggering isomiR-SEA run parameters even in post-analysis, when the expression levels are already computed. Among these parameters, for example, the difference in length between miRNA and tag. This threshold, set to 4, caused the removal of those tags with size shorter or longer than miRNA sequence length ±4. It is worth noting however that this threshold can be modified to match specific user requirements.

Referring to **Sample T27N**, all the tools have been able to attribute to miR-1244 those reads characterized by the presence of a complete seed sequence with exception of miRanalyzer that discarded those alignments having different nucleotides inserted in tag 5’-end, even in the presence of a complete seed sequence. On the other hand deletions in tag 3’-end in presence of complete seeds did not caused tags to be discarded. Furthermore, the first two tags characterized by the lack of a complete seed (reported with red background) are detected by all isomiR-SEA competitor tools. isomiR-SEA instead, being implemented to report tags alignments characterized by the presence of a complete seed sequence, discarded the last three alignments that present mismatches or deletions in the seed. The last red alignment highlights miRExpress sensitivity to mismatches: The tag has been indeed discarded because of a mismatch in the 5’-end.

In **Sample T04N** it is possible to note how tags discarded by isomiR-SEA, because they are missing the complete seed sequence, are instead identified by competitor tools even in presence of only two seed nucleotides. miRanalyzer discarded, also in this case, tags with nucleotides inserted in 5’-end even if characterized by a complete seed sequence. miRExpress as well as miRDeep2, reported all the tags containing seed sequence with exception of the fifth, probably because of the high number of mismatches encountered during its alignment on the whole miRNA sequence.

In **Sample B05N** miRExpress filtered out 5 out of 6 tags harbouring the seed because of a high number of mismatches. However, being these mismatches localized in the 3’-end of tag sequences, they do not account for the loss of interaction sites. As a consequence they cannot be considered responsible for changes in miRNA regulative behaviour and so could be counted, more properly, among miR-1244 supporting tags. Conversely, miRDeep2 detected all the alignments characterized by a complete seed sequence. The two green alignments in position one and three are detected by isomiR-SEA tool but then filtered out because their alignment size was shorter than the aforementioned threshold of 4 nucleotides. Indeed miRNA length is equal to 26 nucleotides whereas tags are respectively 21 and 20 nucleotides long.

However, being isomiR-SEA able (differently from the compared tools) to report in detail during all the alignment phases encountered matches/mismatches and insertions/deletions and to annotate the identified isomiRs, the combinations among them and the conserved interaction sites, the users can select: i) Which tags filter out, ii) which kind of isomiRs consider for miRNA expression evaluation and iii) how to group the data and perform statistics on the basis of the observed results.

All the alignments of **Sample K07N** are discarded by isomiR-SEA because tags missed the seed. Whereas concerning **Sample B20N**, two tags harbouring the seed have been detected by isomiR-SEA even in presence of SNPs.

**Fig. 11:**
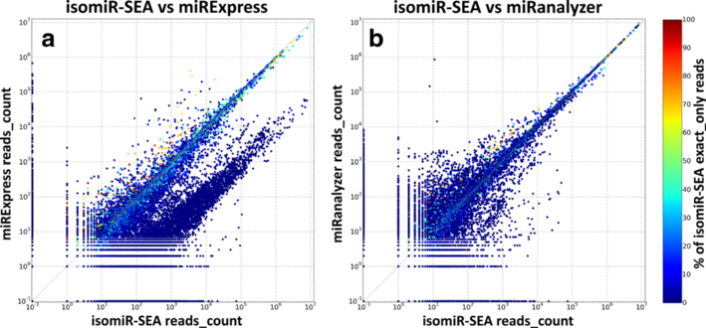
Logarithmic cross-correlation values. Subfigures (**a**) and (**b**) report respectively on the LCCs calculated for isomiR-SEA:miRExpress and for isomiR-SEA:miRanalyzer reads counts. The color bar depicts the percentages of miRNA exact mapped tags detected by isomiR-SEA tool. This color annotation is relative to isomiR-SEA tool only

Differences in the results provided by the compared tools on Li and colleagues dataset, have been further investigated performing cross-correlation analyses. Subfigures 11.a and 11.b report, respectively for isomiR-SEA:miRExpress and isomiR-SEA:miRanalyzer, the logarithmic cross-correlation (*LCC*) of the different miRNAs reads counts detected by each couple of tools. For the sake of clarity, the point (10^−1^,10^−1^) corresponds in the plots of Fig. 11 to the (0,0) coordinate. Each dot, representing the reads counts of a miRNA in a specific sample, assumes near blue colors if the percentage of isomiR-SEA exact mapped reads accounting for its expression is low, whereas reddish colors represent higher percentages. Note that this color annotation is relative to isomiR-SEA tool only because the other tools do not provide this kind of information. The different values in the plots can be further explained in terms of coordinates by sectioning the area in five main portions: i) Dots located on the x-axis account for reads assigned to a given miRNA by *X-tool* that are not assigned by *Y-tool*; ii) dots placed on the y-axis represent reads mapped on a given miRNA only by *Y-tool*; iii) the 45° line reports those miRNAs identified as equally expressed by the two compared tools; iv) the area above the 45° line includes all the miRNAs detected as more expressed by *Y-tool* with respect to *X-tool*; v) the zone under the 45° line reports on those miRNAs detected as more expressed by *X-tool* with respect to *Y-tool*.

With respect to Fig. 11a, isomiR-SEA results account for a relevant number of miRNAs characterized by expression levels two order of magnitude higher than those provided by miRExpress tool. These miRNAs, detected as more expressed by isomiR-SEA tool, are supported by reads harbouring in their sequences the seed, but in the most of the cases present sequence variations with respect to the exact miRNA sequence. They are evaluated as isomiRs by isomiR-SEA. Otherwise, probably because of the high number of mismatches detected with respect to the miRNA sequence, they are not considered by miRExpress due to the inability proper of this tool to detect iso-SNP variants (as showed in Fig. 10).

The *LCC* relative to miRanalyzer and isomiR-SEA read counts are reported in Fig. 11b. Differently from what was previously highlighted in relation to miRExpress and isomiR-SEA comparison, it is not possible here to detect a significant imbalance in the expression of the miRNAs detected by the two tools on the considered dataset. However miRanalyzer produces slightly higher expression levels because, as shown in the analysis reported in Fig. 10, it counts among miRNAs supporting tags also sequences characterized by the absence of seed sequence.

It is worth noting that, in the reported analysis, all the isomiRs were counted by isomiR-SEA in order to evaluate the miRNAs expression, filtering out only those tags with an incomplete seed sequence. However, differently from miRanalyzer and miRExpress, the user can decide to weight the different isomiRs in the expression evaluation or totally exclude some of them. Moreover, the prevalence of blue dots accounts for reads not exactly attributed to miRNAs and probably deriving from isomiRs. Thus, in order to discriminate between the different isomiRs and miRNAs and to correctly evaluate the conservation of miRNA-mRNA interaction sites and, as a consequence, the miRNA regulatory activity, it is necessary to use a tool able to provide accurate information about tags mapping.

Different miRNAs expression levels can be furthermore pointed out when the couple miRExpress:miRanalyzer LCCs is considered, as shown in Additional file 1: Text S4. In this case, it is not possible provide information about isomiRs and thus the tags supporting the different miRNAs have been considered as the exact miRNAs sequence and tags with mismatches in the seed have been also kept. The same analysis is reported in Additional file 1: Text S5 for Somel dataset.

### Computational costs

**Fig. 12:**
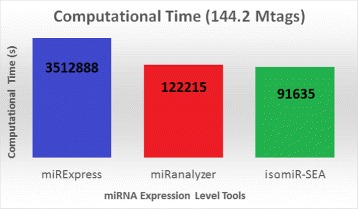
Computational times comparisons. In Figure are reported, for the different tools on x-axis, the overall times required for the analysis of three miRNA-Seq samples

Figure 12 reports the computational times required respectively by miRanalyzer, miRExress and isomiR-SEA, for the analysis of both Somel [48] and Li [49] datasets. Tests have been performed on a Symmetric Multiprocessing (SMP) architecture, namely a 4 + 4 Intel(R) Core(TM) i7 CPUs 920 @2.67 GHz machine, on which no other process was running. For both miRanalyzer and miRExpress default parameters have been set. isomiR-SEA run has been executed instead by adopting the parameters values used for the previously reported analyses. The table of counts have been selected as input for the three compared programs and the computational times have been recorded taking advantage of the bash program *time*.

As it is possible to note from Fig. 12 the most expensive tool, from a computational point of view, is miRExpress that required about 976 h to complete the test. Computational times significantly lower can be pointed out for miRanalyzer, that took about 34 h to finish the analysis. Further lower computational costs have been observed for isomiR-SEA that required about 25 h of computation.

## Conclusions

In this paper we proposed a novel methodology and tool, named isomiR-SEA, able to provide users with a complete and accurate picture of the miRNAs, isomiRs and conserved miRNA:mRNA interaction sites characterizing the tissue under examination. Differently from state of the art tools that rely on general purpose alignment algorithms, isomiR-SEA is built on top of miRNAs specific features, among them, the seed sequence. Its work-flow basically consists in miRNAs seed sequence match followed by a series of ungapped extension in both 5’ and 3’ directions. Being able to keep track of all the performed alignment steps, the tool can evaluate all miRNAs variants, namely isomiRs, and to characterize miRNAs interaction sites.

Concerning isomiRs, isomiR-SEA provides the expression levels of *iso_5p*, *iso_3p*, *iso_snp*, *iso_multi_snp* or combinations of them and allows to distinguish which among *supplementary*, *offset* and *central* interaction sites are conserved in the tags.

isomiR-SEA provides as output two main types of files: The first contains information organized from a tag point of view whereas the second reports results sorted according to the specific miRNA. The proper format of both of them allows to easily process data for i) performing further statistical analyses, ii) filtering specific isomiRs, iii) implementing an isomiR-weighted expression analysis of the detected miRNAs.

Tests performed on the labelled data by Somel and colleagues [48] and Li et al. [49] allowed to highlight isomiR-SEA potential in extracting novel information related to well known miRNAs behaviours as well as its possible applications in different miRNAs studies, such as multi-species, developmental or cancer studies. isomiR-SEA results have been moreover compared to those provided by two well known tools, i.e. miRanalyzer and miRExpress, to prove the proper advantages of a miRNA specific alignment procedure. Finally the reduced computational costs make isomiR-SEA tool a viable solution for the analysis of large dataset and, as a consequence, a great alternative to design wide miRNAs studies.

## Availability and requirements

**Project name:** isomiR-SEA**Project home page:**http://eda.polito.it/isomir-sea**Operating systems:** Linux, Windows, Mac OS X**Programming language:** C, C++**License:** freely available for academic purposes

## Additional file

Additional file 1 **Supplementary Material.** This is a composed pdf file containing supplementary discussions, tables, and figures related to validation tests performed using isomiR-SEA. (PDF 4050 kb)
