# Supplementary material for: isomiR-SEA: an RNA-Seq analysis tool for miRNAs/isomiRs expression level profiling and miRNA-mRNA interaction sites evaluation
Source: BMC Bioinformatics. 2016 Mar 31;17:148. doi: 10.1186/s12859-016-0958-0 (PMC4815201; doi:10.1186/s12859-016-0958-0)
Supplement: Supplementary file 1 — Supplementary Material. This is a composed pdf file containing supplementary discussions, tables, and figures related to validation tests performed using isomiR-SEA. (PDF 4050 kb) [file 12859_2016_958_MOESM1_ESM.pdf]

Supplementary Material for "isomiR-SEA: An RNA-Seq analysis tool for miRNAs/isomiRs expression level profiling and miRNA-mRNA interaction sites evaluation"

Gianvito Urgese [1], Giulia Paciello [1], Andrea Acquaviva [1] and Elisa Ficarra [1]

[1] *Dept. of Control and Computer Engineering DAUIN, Politecnico di Torino, C.so Duca degli Abruzzi 24, Turin, 10129, Italy*

(Dated: February 11, 2016)

TEXT S1

The choice of considering as input sequences those reads trimmed by imposing a maximum number of mismatches equal to 3 is due to the fact that not remarkable differences have been pointed out when a number of mismatches equal to 2 was imposed. Data relative to this study are reported in Table S1 in relation to Somel dataset and in Tables S2 and S3 for Li dataset. Table S1 reports, for the different samples indicated with their GEO accession numbers in the first column, the age of the individual (days, years), a unique identifier for the sample (Sample), the number of reads of the dataset (total_reads), the number of reads and tags after adapter removal with 2 mismatches (Adapter2: reads, tags), the number of reads and tags after adapter removal with 3 mismatches (Adapter3: reads, tags), the number of reads and tags mapped on hsa or hsa-mml miRNA sequences after adapter removal with 3 mismatches (Adapter3: Mapped on hsa: reads_h, tags_h; Adapter3: Mapped on hsa-mml: reads_h-m, tags_h-m). Tables S2 and S3 report, for the different samples indicated with their GEO accession numbers in the first column, the age of the individual (years), a code related to the pathology from which the sample derives (TNM), a unique identifier for the sample (Sample), the number of reads of the dataset (total_reads), the number of reads and tags after adapter removal with 2 mismatches (Adapter2: reads, tags), the number of reads and tags after adapter removal with 3 mismatches (Adapter3: reads, tags), the number of reads and tags mapped on miRBase release 21 and 11 after adapter removal with 3 mismatches (Adapter3: Mapped on db21: reads_21, tags_21; Adapter3: Mapped on db11: reads_11, tags_11). The differences between the average number of reads obtained when trimming the adapter by allowing 2 and 3 mismatches are respectively equal to 4198 for Somel and 680 for Li dataset.

					Adapter 2		Adapter 3					
									Mapped on hsa		Mapped on hsa-mml	
**GEO**	**days**	**years**	**Sample**	**total_reads**	**reads**	**tags**	**reads**	**tags**	**reads_h**	**tags_h**	**reads_h-m**	**tags_h-m**
GSM450597	2	0	hsa_01	6.75E+06	5.67E+06	1.60E+06	5.67E+06	1.29E+06	3.97E+06	8.91E+02	3.94E+06	9.73E+02
GSM450598	34	0	hsa_02	7.30E+06	6.69E+06	1.96E+06	6.69E+06	1.65E+06	4.41E+06	9.79E+02	4.38E+06	1.08E+03
GSM450599	2	8	hsa_03	5.48E+06	4.87E+06	1.76E+06	4.87E+06	1.53E+06	2.82E+06	9.47E+02	2.80E+06	1.04E+03
GSM450600	204	0	hsa_04	8.41E+06	8.15E+06	1.78E+06	8.15E+06	1.52E+06	5.98E+06	1.07E+03	5.93E+06	1.18E+03
GSM450601	112	53	hsa_05	9.81E+06	9.33E+06	3.94E+06	9.32E+06	3.52E+06	4.68E+06	1.08E+03	4.61E+06	1.18E+03
GSM450602	152	25	hsa_06	8.94E+06	8.42E+06	2.97E+06	8.42E+06	2.64E+06	4.66E+06	1.03E+03	4.62E+06	1.14E+03
GSM450603	0	98	hsa_07	5.76E+06	5.61E+06	1.39E+06	5.61E+06	1.13E+06	4.04E+06	9.64E+02	3.99E+06	1.05E+03
GSM450604	0	80	hsa_08	5.82E+06	5.70E+06	1.37E+06	5.69E+06	1.13E+06	4.14E+06	9.77E+02	4.12E+06	1.07E+03
GSM450605	360	13	hsa_09	8.54E+06	8.21E+06	2.74E+06	8.21E+06	2.45E+06	4.76E+06	1.07E+03	4.72E+06	1.17E+03
GSM450606	34	0	hsa_10	7.55E+06	7.28E+06	1.50E+06	7.28E+06	1.28E+06	5.48E+06	1.04E+03	5.45E+06	1.13E+03
GSM450607	4	0	hsa_11	7.94E+06	7.69E+06	1.56E+06	7.69E+06	1.29E+06	5.93E+06	1.02E+03	5.90E+06	1.12E+03
GSM450608	0	98	hsa_12	8.37E+06	7.82E+06	2.45E+06	7.80E+06	2.07E+06	4.83E+06	1.10E+03	4.70E+06	1.20E+03
GSM450609	0	88	hsa_13	9.24E+06	9.03E+06	2.31E+06	9.02E+06	1.99E+06	6.19E+06	1.04E+03	6.13E+06	1.15E+03
GSM450610	0	66	hsa_14	8.52E+06	8.21E+06	1.89E+06	8.21E+06	1.63E+06	5.91E+06	1.06E+03	5.86E+06	1.16E+03
GSM450611	16	0	mml_01	1.13E+07	1.10E+07	2.48E+06	1.10E+07	2.24E+06	7.27E+06	8.24E+02	7.23E+06	1.02E+03
GSM450612	20	0	mml_02	1.15E+07	1.12E+07	2.54E+06	1.12E+07	2.26E+06	7.39E+06	8.05E+02	7.35E+06	9.84E+02
GSM450613	207	0	mml_03	1.19E+07	1.17E+07	2.80E+06	1.16E+07	2.49E+06	7.49E+06	7.80E+02	7.44E+06	9.66E+02
GSM450614	9	2	mml_04	1.05E+07	1.02E+07	1.96E+06	1.02E+07	1.74E+06	7.22E+06	7.67E+02	7.18E+06	9.50E+02
GSM450615	104	9	mml_05	1.07E+07	1.04E+07	2.20E+06	1.04E+07	1.96E+06	6.98E+06	7.63E+02	6.95E+06	9.49E+02
GSM450616	74	22	mml_06	1.18E+07	1.15E+07	2.93E+06	1.15E+07	2.60E+06	7.07E+06	7.86E+02	7.03E+06	9.59E+02
GSM450617	0	28	mml_07	1.02E+07	9.84E+06	2.09E+06	9.84E+06	1.85E+06	6.65E+06	7.51E+02	6.62E+06	9.19E+02
GSM450618	16	0	mml_08	5.84E+06	5.72E+06	2.92E+05	5.72E+06	2.92E+05	4.97E+06	7.07E+02	4.96E+06	8.75E+02
GSM450619	153	0	mml_09	5.22E+06	5.17E+06	2.27E+05	5.17E+06	2.27E+05	4.50E+06	6.60E+02	4.50E+06	8.04E+02
GSM450620	310	0	mml_10	5.26E+06	5.21E+06	2.37E+05	5.21E+06	2.37E+05	4.51E+06	6.62E+02	4.50E+06	8.04E+02
GSM450621	27	4	mml_11	5.63E+06	5.55E+06	1.97E+05	5.55E+06	1.97E+05	4.96E+06	6.64E+02	4.95E+06	7.99E+02
GSM450622	91	20	mml_12	5.74E+06	5.65E+06	2.07E+05	5.65E+06	2.07E+05	5.08E+06	6.54E+02	5.06E+06	7.98E+02
GSM450623	0	28	mml_13	5.58E+06	5.51E+06	2.17E+05	5.51E+06	2.17E+05	4.93E+06	6.75E+02	4.92E+06	8.18E+02

TABLE S1: hsa-mml specs

					Adapter 2		Adapter 3					
									Mapped on db21		Mapped on db11	
**GEO**	**years**	**TNM**	**Sample**	**total_reads**	**reads**	**tags**	**reads**	**tags**	**reads_21**	**tags_21**	**reads_11**	**tags_11**
GSM602577	32	T1N0M0	K01N	1.44E+07	1.37E+07	1.06E+06	1.37E+07	1.06E+06	5.72E+06	1.09E+03	5.57E+06	4.38E+02
GSM602578	32	T1N0M0	K01C	1.43E+07	1.39E+07	1.10E+06	1.39E+07	1.10E+06	6.43E+06	1.07E+03	6.22E+06	4.19E+02
GSM602579	36	T2N0M0	K02N	1.43E+07	1.38E+07	1.44E+06	1.38E+07	1.44E+06	6.79E+06	1.08E+03	6.73E+06	4.24E+02
GSM602580	36	T2N0M0	K02C	1.49E+07	1.43E+07	1.17E+06	1.43E+07	1.17E+06	7.11E+06	1.04E+03	7.04E+06	4.08E+02
GSM602581	41	T2N0M0	K03N	1.79E+07	1.68E+07	2.60E+06	1.68E+07	2.60E+06	3.18E+06	9.75E+02	3.09E+06	4.14E+02
GSM602582	41	T2N0M0	K03C	1.76E+07	1.72E+07	1.65E+06	1.72E+07	1.65E+06	8.17E+06	1.13E+03	8.08E+06	4.35E+02
GSM602583	40	T1N0M0	K06N	1.73E+07	1.69E+07	2.00E+06	1.69E+07	2.00E+06	5.32E+06	1.15E+03	5.26E+06	4.56E+02
GSM602584	40	T1N0M0	K06C	1.51E+07	1.45E+07	1.73E+06	1.45E+07	1.73E+06	4.10E+06	1.07E+03	4.04E+06	4.32E+02
GSM602585	27	T1N0M0	K07N	1.47E+07	1.38E+07	1.62E+06	1.38E+07	1.62E+06	4.36E+06	1.06E+03	4.33E+06	4.16E+02
GSM602586	27	T1N0M0	K07C	1.41E+07	1.35E+07	1.79E+06	1.35E+07	1.79E+06	4.04E+06	1.04E+03	3.99E+06	4.36E+02
GSM602587	52	T2N0M0	K27N	1.87E+07	1.81E+07	1.73E+06	1.81E+07	1.73E+06	9.24E+06	1.09E+03	8.99E+06	4.33E+02
GSM602588	52	T2N0M0	K27C	1.92E+07	1.88E+07	2.17E+06	1.88E+07	2.17E+06	8.26E+06	1.05E+03	8.20E+06	4.26E+02
GSM602589	40	T1N0M0	K38N	1.91E+07	1.81E+07	2.39E+06	1.81E+07	2.39E+06	7.95E+06	1.13E+03	7.77E+06	4.48E+02
GSM602590	40	T1N0M0	K38C	1.89E+07	1.82E+07	2.25E+06	1.82E+07	2.25E+06	7.02E+06	9.29E+02	6.83E+06	3.84E+02
GSM602591	58	T2N0M0	K39N	1.88E+07	1.81E+07	2.44E+06	1.81E+07	2.44E+06	5.48E+06	1.03E+03	5.40E+06	4.24E+02
GSM602592	58	T2N0M0	K39C	1.96E+07	1.88E+07	2.66E+06	1.88E+07	2.66E+06	4.91E+06	1.05E+03	4.87E+06	4.16E+02
GSM602593	48	T2N0M0	K44N	1.87E+07	1.80E+07	2.18E+06	1.80E+07	2.18E+06	8.36E+06	1.12E+03	8.20E+06	4.50E+02
GSM602594	48	T2N0M0	K44C	1.93E+07	1.86E+07	2.51E+06	1.86E+07	2.51E+06	5.92E+06	9.93E+02	5.88E+06	4.25E+02
GSM602595	52	T4N0M0	K55N	1.78E+07	1.75E+07	2.08E+06	1.75E+07	2.08E+06	9.04E+06	1.08E+03	8.90E+06	4.23E+02
GSM602596	52	T4N0M0	K55C	1.77E+07	1.73E+07	2.31E+06	1.73E+07	2.31E+06	7.40E+06	1.11E+03	7.25E+06	4.47E+02
GSM785413	66	T1N0M0	B04C	1.89E+07	1.84E+07	2.60E+06	1.84E+07	2.47E+06	4.74E+06	1.03E+03	4.68E+06	4.19E+02
GSM785414	66	T1N0M0	B04N	1.40E+07	1.38E+07	1.11E+06	1.38E+07	9.89E+05	9.69E+06	7.84E+02	9.64E+06	3.86E+02
GSM785415	64	T2N0M0	B05C	1.76E+07	1.72E+07	2.03E+06	1.72E+07	1.90E+06	6.91E+06	1.07E+03	6.82E+06	4.36E+02
GSM785416	64	T2N0M0	B05N	1.90E+07	1.86E+07	2.41E+06	1.86E+07	2.32E+06	6.40E+06	1.08E+03	6.32E+06	4.33E+02
GSM785417	40	T4aN0M0	B06C	1.91E+07	1.90E+07	2.63E+06	1.90E+07	2.54E+06	6.54E+06	1.04E+03	6.48E+06	4.30E+02
GSM785418	40	T4aN0M0	B06N	1.56E+07	1.55E+07	1.58E+06	1.55E+07	1.48E+06	9.06E+06	9.35E+02	8.99E+06	4.09E+02
GSM785419	72	T1N0M0	B07C	1.87E+07	1.85E+07	2.69E+06	1.85E+07	2.60E+06	5.19E+06	1.15E+03	5.13E+06	4.59E+02

TABLE S2: genito 1 part specs

					Adapter 2		Adapter 3					
									Mapped on db21		Mapped on db11	
**GEO**	**years**	**TNM**	**Sample**	**total_reads**	**reads**	**tags**	**reads**	**tags**	**reads_21**	**tags_21**	**reads_11**	**tags_11**
GSM785420	72	T1N0M0	B07N	1.60E+07	1.59E+07	1.56E+06	1.59E+07	1.44E+06	8.36E+06	8.92E+02	8.31E+06	4.07E+02
GSM785421	66	T4N0M0	B08C	1.78E+07	1.71E+07	2.11E+06	1.71E+07	1.98E+06	5.90E+06	1.06E+03	5.83E+06	4.33E+02
GSM785422	66	T4N0M0	B08N	1.43E+07	1.41E+07	1.29E+06	1.41E+07	1.16E+06	9.52E+06	9.04E+02	9.44E+06	3.98E+02
GSM785423	65	T4aN0M0	B10C	9.71E+06	9.40E+06	3.96E+05	9.39E+06	3.92E+05	1.48E+06	6.91E+02	1.45E+06	3.35E+02
GSM785424	65	T4aN0M0	B10N	1.14E+07	1.11E+07	1.81E+05	1.11E+07	1.77E+05	7.88E+06	6.71E+02	7.86E+06	3.52E+02
GSM785425	55	T2N0M0	B01C	1.85E+07	1.80E+07	2.09E+06	1.80E+07	1.98E+06	9.10E+06	1.48E+03	8.99E+06	5.03E+02
GSM785426	55	T2N0M0	B01N	1.78E+07	1.72E+07	1.76E+06	1.72E+07	1.63E+06	7.96E+06	1.30E+03	7.84E+06	4.77E+02
GSM785427	72	T4NxMx	B09C	1.31E+07	1.28E+07	1.37E+06	1.28E+07	1.35E+06	3.78E+06	1.11E+03	3.79E+06	4.24E+02
GSM785428	72	T4NxMx	B09N	1.00E+07	9.85E+06	5.28E+05	9.85E+06	5.14E+05	7.02E+06	7.96E+02	6.99E+06	3.79E+02
GSM785429	42	T2N0M0	B02C	2.01E+07	1.98E+07	2.37E+06	1.98E+07	2.26E+06	6.85E+06	1.13E+03	6.77E+06	4.37E+02
GSM785430	42	T2N0M0	B02N	1.77E+07	1.73E+07	1.73E+06	1.73E+07	1.61E+06	8.99E+06	1.05E+03	8.90E+06	4.30E+02
GSM785431	53	T4N0M0	B03C	1.87E+07	1.83E+07	2.25E+06	1.83E+07	2.14E+06	7.69E+06	1.05E+03	7.63E+06	4.19E+02
GSM785432	53	T4N0M0	B03N	1.93E+07	1.89E+07	2.37E+06	1.89E+07	2.24E+06	5.90E+06	9.84E+02	5.85E+06	4.17E+02
GSM785433	41	T1N0M0	T03C	1.19E+07	1.17E+07	1.46E+06	1.17E+07	1.45E+06	2.94E+06	1.12E+03	2.89E+06	4.82E+02
GSM785434	41	T1N0M0	T03N	1.16E+07	1.14E+07	1.63E+06	1.14E+07	1.62E+06	1.49E+06	9.63E+02	1.47E+06	4.69E+02
GSM785435	48	T1N0M0	T04C	1.99E+07	1.99E+07	1.48E+06	1.99E+07	1.44E+06	1.07E+07	1.24E+03	1.05E+07	4.47E+02
GSM785436	48	T1N0M0	T04N	2.36E+07	2.36E+07	7.21E+05	2.36E+07	6.91E+05	1.67E+07	1.02E+03	1.67E+07	4.03E+02
GSM785437	27	T1N0M0	T05C	2.42E+07	2.42E+07	1.19E+06	2.42E+07	1.17E+06	1.83E+07	1.54E+03	1.81E+07	5.27E+02
GSM785438	27	T1N0M0	T05N	2.46E+07	2.46E+07	9.77E+05	2.46E+07	9.54E+05	1.97E+07	1.57E+03	1.96E+07	5.35E+02
GSM785439	48	T1N0M0	T06C	2.44E+07	2.44E+07	1.13E+06	2.44E+07	1.10E+06	1.63E+07	1.64E+03	1.61E+07	5.45E+02
GSM785440	48	T1N0M0	T06N	1.97E+07	1.97E+07	6.73E+05	1.97E+07	6.28E+05	9.64E+06	1.43E+03	9.61E+06	5.24E+02
GSM785441	46	T1N0M0	T01C	1.52E+07	1.47E+07	1.85E+06	1.47E+07	1.76E+06	3.46E+06	1.27E+03	3.38E+06	4.98E+02
GSM785442	46	T1N0M0	T01N	1.50E+07	1.45E+07	1.63E+06	1.45E+07	1.53E+06	3.14E+06	1.09E+03	3.09E+06	4.50E+02
GSM785443	30	T1N0M0	T07C	2.45E+07	2.44E+07	6.12E+06	2.44E+07	6.04E+06	1.01E+07	1.22E+03	9.98E+06	4.42E+02
GSM785444	30	T1N0M0	T07N	2.06E+07	2.05E+07	5.41E+06	2.05E+07	5.31E+06	5.65E+06	1.10E+03	5.61E+06	4.19E+02
GSM785445	27	T1N0M0	T02C	1.49E+07	1.42E+07	2.31E+06	1.42E+07	2.25E+06	1.94E+06	1.18E+03	1.90E+06	4.85E+02
GSM785446	27	T1N0M0	T02N	1.46E+07	1.41E+07	4.39E+06	1.41E+07	4.34E+06	2.45E+06	1.20E+03	2.11E+06	4.83E+02

TABLE S3: genito 2 part specs

TEXT S2

IsomiRs as well as exact mapped reads trends, highlighted in the main document using percentage values, can be also appreciated in terms of absolute reads counts as shown in Figure S1 in relation to miR-181a-3p.
IsomiRs are depicted with different colors according to the legend of Table 1 whereas the black, brown, red and blue lines report respectively on isomiR-SEA, miRanalyzer, miRExpress and Somel detected reads counts.
A decreasing number of reads accounting for exact **miR-181a-3p** can be detected in humans at increasing ages with a minimum number of 29 reads reached in the Sample h08 at 80 years. Furthermore the same representation allows to point out an increasing trend in the number of detected *iso_3p* and *iso_5p-iso_3p* during human ageing with a maximum reached at 98 years in Sample h07 and in its replicate h12.

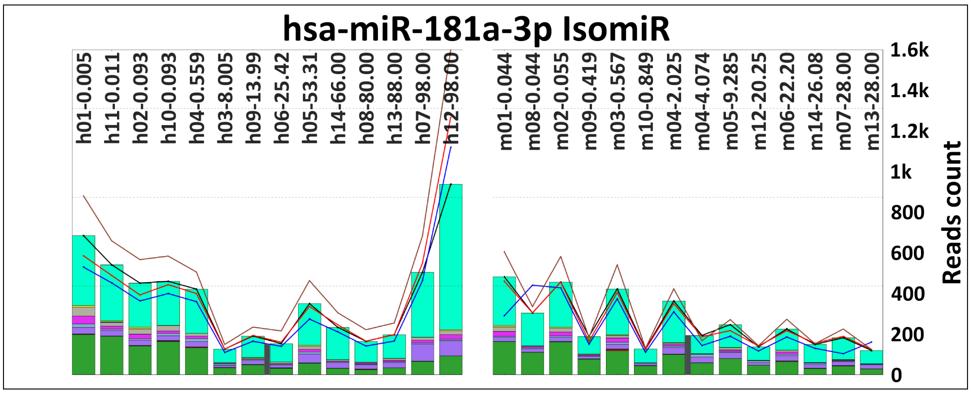

FIG. S1: **hsa-miR-181a-3p isomiRs**. In Figure are reported for human miR-181a-3p on the y-axis the reads counts associated to the different detected isomiRs in the samples under examination (x-axis). The different samples are labelled with a unique identifier in the format speciesnumsample-age: The species can be h for humans or m for macaques, numsample ranges from 01 to 14 for both the species and the age is in the form of years. Five trend curves account respectively for virtual (black dashed), isomiR-SEA (black continue), Somel (blue continue), miRExpress (red continue) and miRanalyzer (brown continue) reads counts.

TEXT S3

In Figures S2, S3 and S4 are reported the expression levels calculated by miRDeep2 for miR-1244 in the five samples (T27N, T04N, B05N, K07N and B20N) from Li dataset.

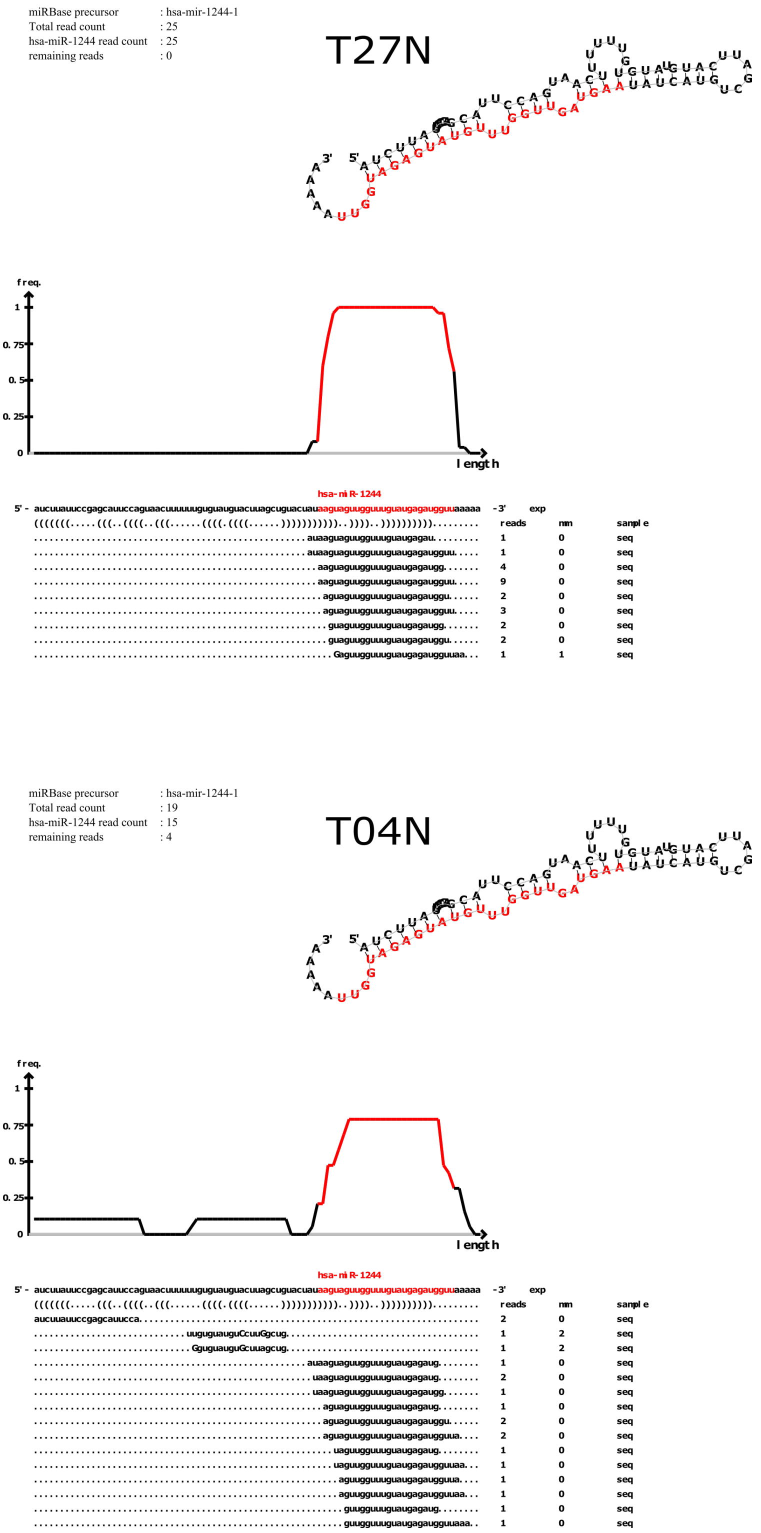

FIG. S2: **miRDeep2 mapping for miR-2144 on samples T27N and T04N.**

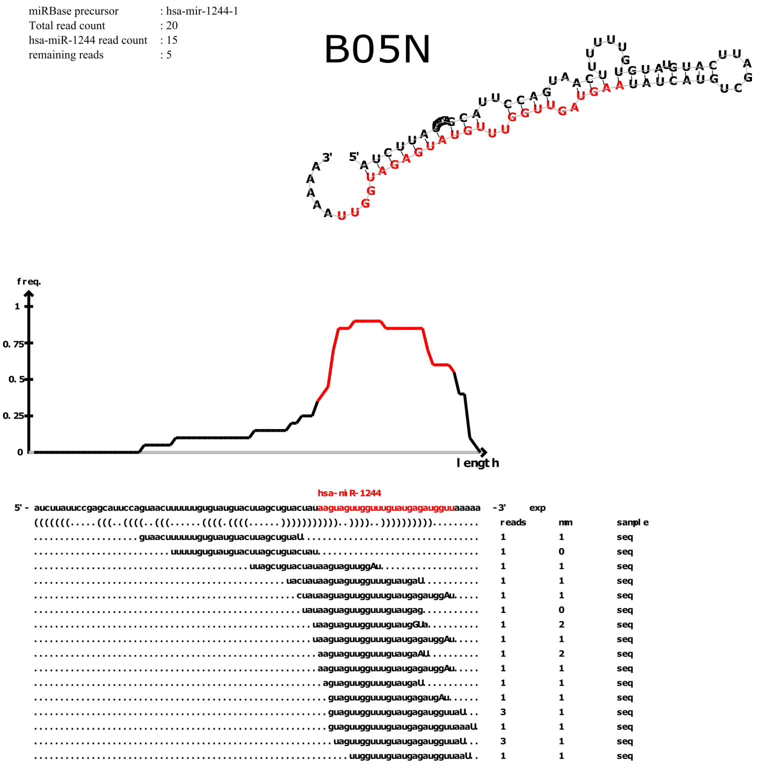

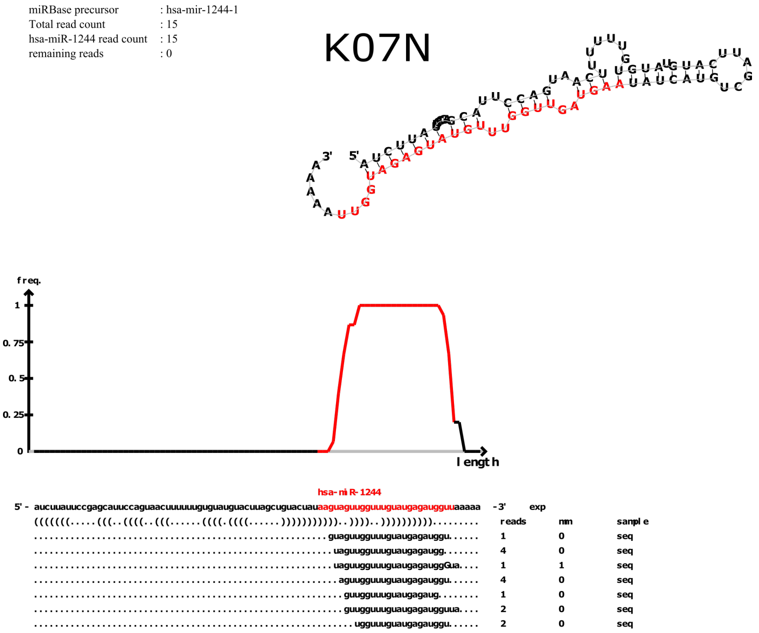

FIG. S3: **miRDeep2 mapping for miR-2144 on samples B05N and K07N**.

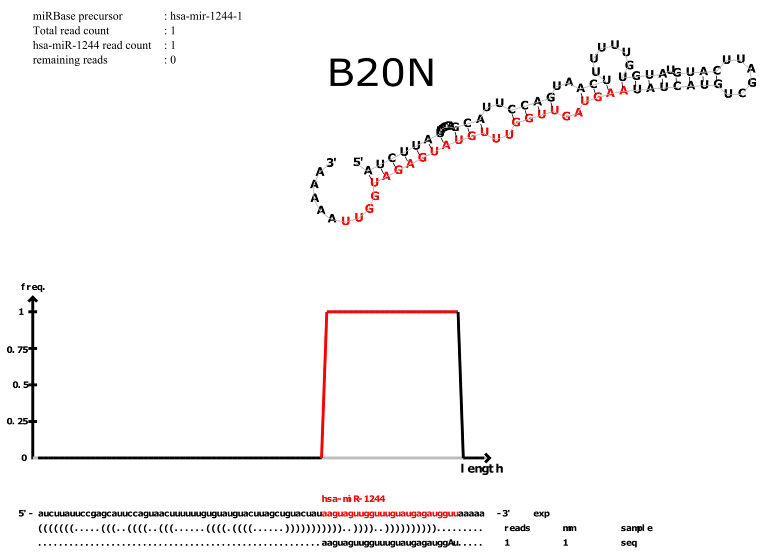

FIG. S4: **miRDeep2 mapping for miR-2144 on sample B20N**.

TEXT S4

Figure S5 reports on miRanalyzer:miRExpress LCC values between reads counts. The predominance of dots under the 45° line accounts for a relevant number of miRNAs detected by miRanalyzer as characterized by a higher expression level with respect to that revealed by the competitor tool. Being both the tools not implemented by considering biological miRNA features such as the seed sequence, the detected expression could be not directly associated to miRNA molecules. Furthermore the absence of information concerning the executed mapping do not allow to distinguish among miRNAs exact mapped reads or reads deriving from isomiRs sequencing.

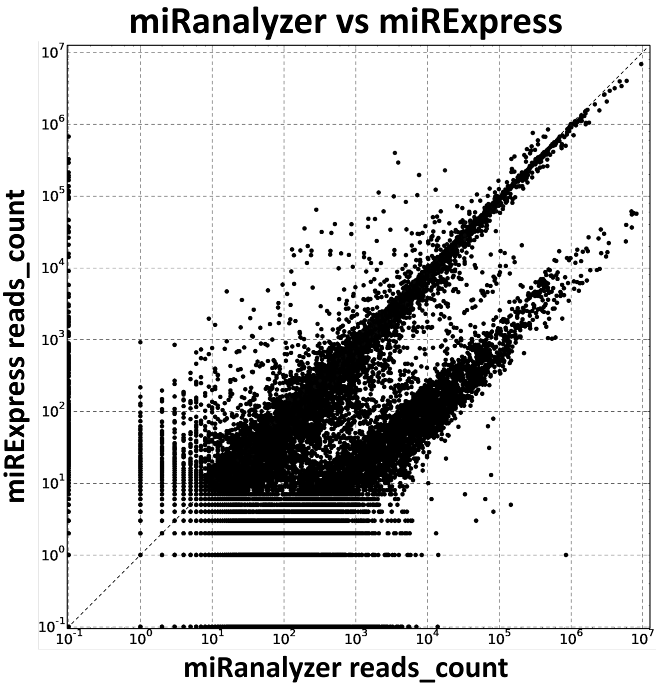

FIG. S5: **Logarithmic cross-correlation values.** In Figure is reported the LCCs calculated for miRanalyzer:miRExpress reads count for the Li dataset.

TEXT S5

Figure S6 A and B report respectively for isomiR-SEA:miRExpress and isomiR-SEA:miRanalyzer, the LCC values from Somel dataset. Figure S7 describes instead miRanalyzer:miRExpress LCCs on the same dataset. The LCC values calculated for miRExpress:isomiR-SEA in Figure S6.A are similar to those from Li dataset. Differently from what observed for Li dataset, it is not possible to detect a significant imbalance in the miRNAs expression levels provided by miRanalyzer and isomiR-SEA tools (Figure S6.B). Finally, in S7, the predominance of dots under the 45° line accounts for a relevant number of miRNAs detected by miRanalyzer as characterized by a higher expression level with respect to that revealed by the competitor tool. This result is concordant to what observed in relation to Li dataset.

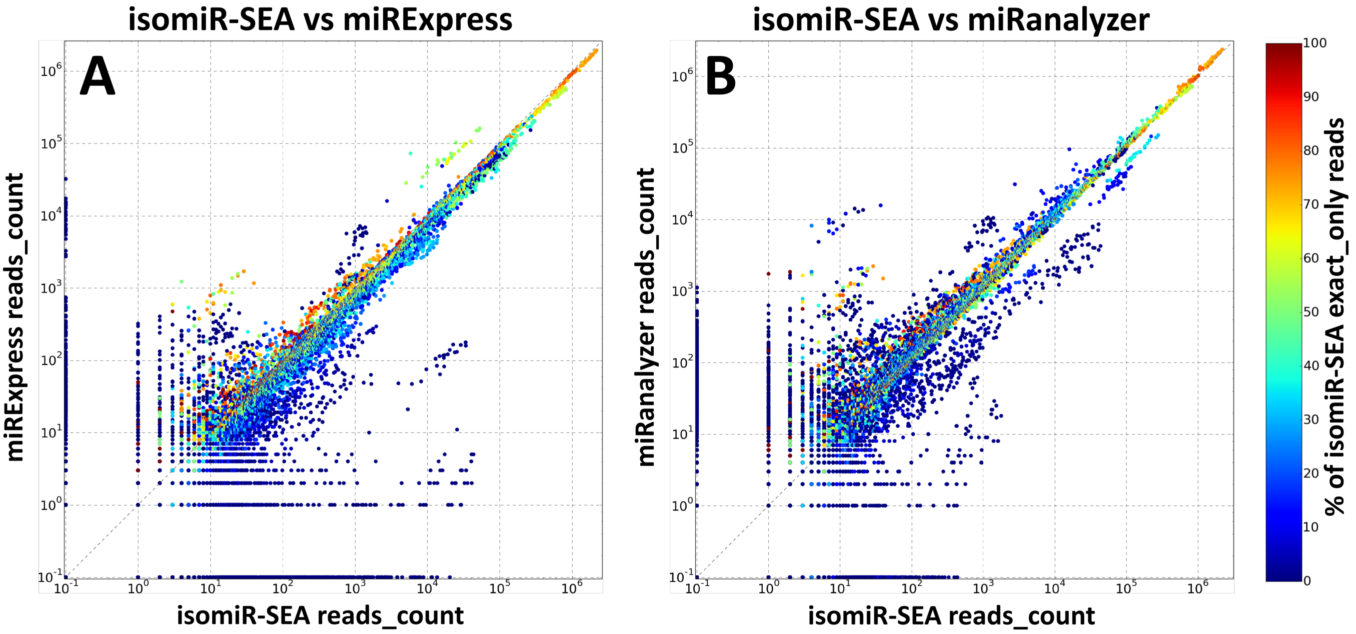

FIG. S6: **Logarithmic cross-correlation values**. Subfigures A and B report respectively on the LCCs calculated for isomiR-SEA:miRExpress and for isomiR-SEA:miRanalyzer reads counts for the Somel dataset. The color bar depicts the percentages of miRNA exact mapped tags detected by isomiR-SEA tool. This color annotation is relative to isomiR-SEA tool only.

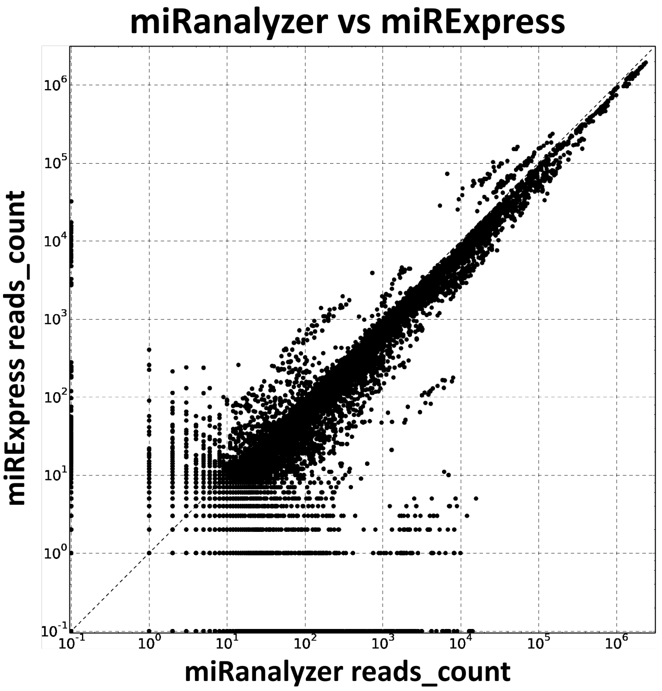

FIG. S7: **Logarithmic cross-correlation values.** In Figure is reported the LCCs calculated for miRanalyzer:miRExpress reads count for the Somel dataset.
